# An Alternative Splice Variant of HIPK2 with Intron Retention Contributes to Cytokinesis

**DOI:** 10.3390/cells9020484

**Published:** 2020-02-20

**Authors:** Veronica Gatti, Manuela Ferrara, Ilaria Virdia, Silvia Matteoni, Laura Monteonofrio, Simona di Martino, Maria Grazia Diodoro, Giuliana Di Rocco, Cinzia Rinaldo, Silvia Soddu

**Affiliations:** 1 Unit of Cellular Networks and Molecular Therapeutic Targets; IRCCS-Regina Elena National Cancer Institute, 00144 Rome, Italy; veronica.gatti@uniroma2.it (V.G.); ilaria.virdia@ifo.gov.it (I.V.); silvia.matteoni@ifo.gov.it (S.M.); laura.monteonofrio@nih.gov (L.M.); giuliana.dirocco@ifo.gov.it (G.D.R.); 2 Institute of Molecular Biology and Pathology (IBPM), National Research Council (CNR), c/o Sapienza University, 00185 Rome, Italy; manu.ferr889@gmail.com; 3 Department of Sciences, University Roma Tre, 00154 Rome, Italy; 4 Pathology Unit, IRCCS-Regina Elena National Cancer Institute, 00144 Rome, Italy; simona.dimartino@ifo.gov.it (S.d.M.); maria.diodoro@ifo.gov.it (M.G.D.)

**Keywords:** HIPK2 isoforms, alternative splicing, abscission, faithful cytokinesis, colorectal cancer, pancreatic cancer

## Abstract

HIPK2 is a DYRK-like kinase involved in cellular stress response pathways, development, and cell division. Two alternative splice variants of HIPK2, HIPK2-FL and HIPK2-Δe8, have been previously identified as having different protein stability but similar functional activity in the stress response. Here, we describe one additional HIPK2 splice variant with a distinct subcellular distribution and functional activity in cytokinesis. This novel splice variant lacks the last two exons and retains intron13 with a stop codon after 89 bp of the intron, generating a short isoform, HIPK2-S, that is detectable by 2D Western blots. RT-PCR analyses of tissue arrays and tumor samples show that HIPK2-FL and HIPK2-S are expressed in normal human tissues in a tissue-dependent manner and differentially expressed in human colorectal and pancreatic cancers. Gain- and loss-of-function experiments showed that in contrast to HIPK2-FL, HIPK2-S has a diffuse, non-speckled distribution and is not involved in the DNA damage response. Rather, we found that HIPK2-S, but not HIPK2-FL, localizes at the intercellular bridge, where it phosphorylates histone H2B and spastin, both required for faithful cell division. Altogether, these data show that distinct human HIPK2 splice variants are involved in distinct HIPK2-regulated functions like stress response and cytokinesis.

## 1. Introduction

HIPK2 (Homeodomain-Interacting Protein Kinase 2) is a Y-regulated S/T kinase [1,2] originally identified for its capacity to interact with homeodomain transcription factors [3]. HIPK2 binds and phosphorylates a broad range of targets and is involved in several signaling pathways, including TP53, WNT/β-Catenin, TGF-β, Hippo, and Interferon [4,5,6,7,8,9,10,11,12,13,14]. HIPK2 gain- and loss-of-function experiments in numerous systems and cell types have revealed its pleiotropic effects as a modulator of even contradictory biological events such as growth arrest and cell death, cell survival and proliferation, and morphogenesis and differentiation [15]. Several post-translational modifications, including phosphorylation, acetylation, SUMO, and Ubiquitin modifications, regulate HIPK2 intracellular localization, stability, and activity and have been proposed to modulate dynamic, tissue-specific, and context dependent activities of the kinase [16,17,18].

Conflicting roles of HIPK2 have also been reported in relation to pathologic conditions, such as cancer and tissue fibrosis. HIPK2’s function as a tumor suppressor has been supported by several pieces of evidence, including the HIPK2 pro-apoptotic and growth-arresting activities through p53-dependent and -independent mechanisms [19,20,21,22,23,24,25,26], the observation that the *Hipk2* gene works as a haploinsufficient tumor suppressor in mice [27], and the identification of a few mechanisms of HIPK2 inactivation in human cancers, such as reduced expression and loss of heterozygosity [28,29,30,31]. In contrast to these observations, HIPK2 has been shown to have prosurvival and proproliferative oncogenic activities in different types of cancers, including a *Drosophila* leukemia model [32,33]. Increased HIPK2 expression with or without gene amplification has been observed in pilocytic astrocytoma [34,35], cervical [36], and renal carcinomas [37], while HIPK2-mediated protection against genotoxic insults has been found in NRF2-overexpressing tumor cells [38]. Strong proproliferative activity of HIPK2 has also been related to kidney and skin fibrosis, in which excessive fibroblast proliferation is coupled with the deposition of extracellular matrix and epithelial-to-mesenchymal transition [39,40].

One additional proliferation-associated role of HIPK2 has been reported in abscission, the final step of cytokinesis, in which the two daughter cells are physically separated [41,42,43]. We have shown that HIPK2 localizes at the intercellular bridge (ICB) connecting the daughter cells, in an Aurora B-dependent manner [44] and contributes to abscission by phosphorylating the extrachromosomal histone H2B at S14 [42,45] and the microtubule-severing enzyme spastin at S268 [46]. Loss of HIPK2 results in cytokinesis failure, the accumulation of tetra and polyploid cells, and the generation of aneuploidy, chromosomal instability, and increased tumorigenicity, supporting a caretaker function [47].

Alternative splicing is a major source for protein diversity in higher eukaryotes [48,49]. Proteins involved in divergent functions, such as H-Ras, Bcl-x, Fas, and the p53 family members, undergo alternative splicing and even generate isoforms with opposing roles [50,51]. The existence of an alternative splice variant of HIPK2, the HIPK2-∆e8 isoform, has been described in colorectal cancer [52]. Compared with the full-length isoform (HIPK2-FL), HIPK2-∆e8 is generated by a partial skipping of exon 8 and lacks 27 amino acids (aa) required for binding with the E3 ubiquitin ligase Siah-1. Thus, HIPK2-∆e8 is more resistant than HIPK2-FL to proteasome digestion; however, no functional difference has been observed, thus far, between these two isoforms [52,53].

Here, we explore the possibility of additional alternative splicing of HIPK2 that might generate isoform(s) with diverse function(s). By a combination of in silico analysis and in vitro experiments, we describe and characterize a novel, short HIPK2 isoform (HIPK2-S) generated by intron retention that specifically contributes to cytokinesis and the prevention of tetraploidization.

## 2. Materials and Methods

### 2.1. Data Source and Bioinformatic Tools

In silico analyses were performed using the University of California Santa Cruz (UCSC) Genome browser platform (https://genome.ucsc.edu/index.html). Interrogations were executed on RefSeq collections, curated and annotated by UCSC and NCBI (National Center for Biotechnology Information) and *Homo sapiens* EST database and ENCODE project track collection. Polyadenylation analyses were also carried out through the UCSC Genome browser platform, interrogating polyA_DB web server (http://exon.umdnj.edu/polya_db/) and PolyA-Seq collection [54], GEO accession number GSE30198. Non-human genomic sequences were extracted from available UCSC genome browser genomes and aligned to the human *HIPK2* gene using pairwise sequence alignment EMBOSS Needle (https://www.ebi.ac.uk/Tools/psa/emboss_needle/).

### 2.2. Cells and Reagents

Human HeLa and HeLa HIPK2-null cells (kindly provided by ML Schmitz, Justus-Liebig-University, Giessen, Germany), HCT116 (kindly provided by B. Vogelstein, Johns Hopkins University School of Medicine, Baltimore, MD, USA), U2OS (kindly provided by F. Moretti, CNR, Rome, Italy), and hTERT-immortalized dermal human fibroblasts (HFs) (kindly provided by F. Loreni, University of Rome Tor Vergata, Rome, Italy) were cultured in DMEM GlutaMAX with 10% heat-inactivated fetal bovine serum (FBS) (Life Technologies, Carlsbad, CA, USA). Cells were routinely tested for mycoplasma contamination. PBMCs were isolated and stimulated to proliferate as described [55]. For EdU (5-ethynyl-2’-deoxyuridine) incorporation and detection, cells were incubated in the presence of 10 μM EdU labeling solution for 3 h, developed according to manufacturing instructions (Click-iT^®^ EdU imaging Kit, Thermo Fisher Scientific, Waltham, MA, USA), and analyzed as described [56]. For DNA damage response, subconfluent cells in 35-mm Petri dishes were incubated in the presence of 0.6 μM doxorubicin (Adriamycin; Sigma-Aldrich, St. Louis, MO, USA). At least 500 cells per sample were counted. Cells were enriched in telophase by treatment with nocodazole (Sigma-Aldrich; 100 ng/mL for 4 h), followed by mitotic shake-off, nocodazole washout, and replating for 90 or 100 min, as described [44].

### 2.3. Patient Specimens

Frozen specimens were from Biobank IRCCS—Regina Elena National Cancer Institute (BBIRE), Rome, Italy. Tumors were staged according to the American Joint Committee on Cancer Staging Manual. The Institutional Ethics Committee (Comitato Etico Centrale I.R.C.C.S. Lazio, Sezione IRCCS I.F.O.—Fondazione G.B. Bietti) approved this study (CE/694/15) and all patients signed their informed consent for participation.

### 2.4. RNA Purification, Reverse Transcriptase RT-PCR, and Quantitative Real-Time qPCR Analyses

Total mRNA from cells was isolated using the RNeasy mini Kit (Qiagen, Hilden, Germany). cDNA was synthesized by M-MLTV RTase and amplified by GoTaq DNA polymerase (Promega, Madison, WI, USA). PCR-amplifications were performed at least in duplicate on two different RNA preparations. Total mRNA from PDAC was extracted by the RNeasy mini kit (Qiagen) after tissue homogenization using a gentleMACS C Tube (Miltenyi BioTec, Bergisch Gladbach, Germany). The RNA template was qualitatively assessed and quantified using a Nanodrop TM 1000 spectrophotometer (Nanodrop Technologies LLC, Thermo Fisher Scientific). cDNA was synthesized by High-Capacity cDNA Reverse Transcription Kit (Applied Biosystems, Foster City, CA, USA). The Human Major Tissue qPCR Panel (cat# HMRT303), containing five identical sets of dried first-strand cDNA from 48 human tissues, and the TissueScan Colon Cancer Tissue qPCR Panel III—Matched Pairs (cat# HCRT303), containing five identical sets of 48 tissues covering four disease stages and normal tissues, were provided by OriGene Technologies, Inc. (Rockville, MD, USA) and analyzed by qPCR following the manufacturer’s instructions. qPCR analyses were carried out using the specific oligonucleotides listed in Table 1. The mRNA expression was measured by real-time qPCR using the SYBR Green DNA master mix (Applied Biosystems, Thermo Fisher Scientific) on a 7500 Real-Time PCR System (Applied Biosystems). All primer sets worked under identical quantitative PCR cycling conditions (1 cycle at 95 °C/10 min, followed by 40 three-segment cycles (95 °C/15 s, 60 °C/35 s, 72 °C/45 s). with similar efficiencies. Relative mRNA expression levels were determined by using the 2^-ΔΔCT^ method, employing *GAPDH* gene expression for data normalization. All reactions were performed in triplicate.

### 2.5. Engineering of Expression Vectors and Transfection

Synthetic sequences of HIPK2-FL (NM_022740) and HIPKS-S were produced and cloned into pUC57 by Eurofins Genomics (Ebersberg, Bayern, Germany). pEGFP-HIPK2-FL and pEGFP-HIPK-S were obtained by subcloning HIPK2-FL and HIPK2-S into pEGFP-c2 (Clontech, Mountain View, CA, USA). Vectors were transfected by using Lipofectamine LTX and Plus reagent (Life Technologies).

### 2.6. RNA Interference

Cells were transfected with 20 nM of siRNA (Table 2) in a single pulse using RNAiMAX reagent (Invitrogen, Thermo Fisher Scientific) according to the manufacturer’s instructions. Red fluorescent oligonucleotides (Block-it, Invitrogen, Thermo Fisher Scientific) were used to evaluate transfection efficiency.

### 2.7. Immunoprecipitation and In Vitro Kinase Assay

GFP-HIPK2-FL, GFP-HIPK2-S, and GFP alone were produced in U2OS cells by transfection. Total cell extracts were prepared 24 h post-transfection, and GFP-fusion proteins were purified by overnight incubation with anti-GFP antibody (Ab)-sepharose beads (rabbit polyclonal ab69314, Abcam, Cambridge, UK) and used as an enzymatic source as described [57]. WB analysis with a different anti-GFP moAb (LGB-1, Abcam) was performed to quantify purified proteins. Kinase assays were performed by incubating immunoprecipitated proteins with MBP in a kinase buffer [Hepes 20 mM pH 7.5, 1 mM DTT, 10 mM MgCl_2_, and 1 mM EGTA] in the presence of γ-^32^P-ATP (Perlkin-Elmer, Waltham, MA, USA) at 30 °C for 30 min. Proteins were resolved on precast NuPAGE 4%–12% gels (Thermo Fisher Scientific) and analyzed by autoradiography.

### 2.8. Mono-Dimensional and 2D Gel Electrophoresis and Western Blotting

For mono-dimensional gels, total cell lysates were prepared in a lysis buffer (50 mM Tris-HCl pH 8.0, 600 mM NaCl, 0.5% sodium deoxycholate, 0.1% SDS, 1% NP40 and 1 mM EDTA) supplemented with protease-inhibitor mix (Roche, Basel, Switzerland) and Halt Phosphatase Inhibitor Cocktail (Life Technologies). As strong extraction condition, cells were lysed directly in hot Laemmli sample buffer. NuPAGE^®^ Novex Bis-Tris Gels (Life Technologies) were used for SDS-PAGE and nitrocellulose membranes (Bio-Rad, Hercules, CA, USA) for protein transfer and immobilization. For 2D-gels, cell pellets were resuspended in 2D lysis buffer (2 M thiourea, 7 M urea, 4% CHAPS, 1% DTT) and briefly sonicated. Lysates were then centrifuged at 18,000× *g* for 10 min at RT and supernatants were transferred to clean tubes. Isoelectric focusing was performed using 70-mm 3–10 NL Immobilized pH gradient (IPG) strips (Bio-Rad) on an electrophoresis unit (Ettan IPGphor 3; GE Healthcare, Chicago, IL, USA). An amount of 125 µL of 2D lysis buffer containing 90–100 µg of each protein sample, with the addition of 1% sulfobetaine SB 3–10, were used for strip passive in-gel rehydration for 12 h and then run as follows: 30 min at 50 V, 30 min at 200 V, voltage gradient 30 min up to 1000 V, 20 min at 1,000 V, voltage gradient 1 h up to 5000 V and 1–2 h at 5000 V. Before performing the second electrophoretic dimension, IPG gel strips were equilibrated for 15 min at RT in 1% DTT in order to reduce the proteins, and sulfhydryl groups were subsequently derivatized using 4% iodoacetamide; both solutions were prepared in (50 mM TRIS, pH 8.8, 6 M urea, 30% glycerol, 2% SDS, and 2% bromophenol blue) for 15 min. Strips were transferred to 1.0-mm-thick 7% polyacrylamide minigels for the second electrophoresis dimension. Gels were run at 120 V for 2 h and transferred onto nitrocellulose membranes (Bio-Rad). The following Abs were employed for WB: anti-α-tubulin moAb (Societa’ Italiana Chimici divisione scientifica s.r.l., Rome, Italy), anti-GFP moAb (Roche), anti-HIPK2 rabbit polyclonal (kindly provided by ML Schmitz) [23]; anti-HIPK2 C-Ter rat moAb (C5C6, kindly provided by ML Schmitz); anti-GAPDH moAb (Santa Cruz Biotechnology, Dallas, TX, USA); anti-HIPK2-S rabbit polyclonal (self-made, see below); HRP-conjugated goat anti-mouse and anti-rabbit secondary Abs (Bio-Rad). Immunoreactivity was determined using the ECL-chemiluminescence or ECL-Prime reactions (GE Healthcare) following the manufacturer’s instructions. Image acquisition was performed by autoradiography or the ChemiDoc (Bio-Rad) imaging system.

### 2.9. Immunofluorescence Microscopy

Cells seeded on poly-L-lysine coated coverslips were fixed as described [46]. The following Abs were employed: anti-phosho-H2B-S14 rabbit moAb (D67H2, Cell Signaling, Danvers, MA, USA); anti-phospho-Spatin-S268 rabbit polyclonal (self-made) [46]; anti-HIPK2 (946, self-made rabbit polyclonal Ab [42]; anti-p-histone-H2B-S14 (Cell Signaling, Danvers, MA, USA); anti-β-tubulin-Cy3 (TUB2.1, Sigma-Aldrich); anti-α-tubulin-FITC (DM1A, Sigma-Aldrich). Secondary FITC- and TRITC-conjugated Abs (Alexa-fluor, Invitrogen). DNA was marked with Hoechst 33342 or DAPI (both from Sigma-Aldrich). Cells were examined with Olympus BX53 microscope (Olympus Life Science). GFP fluorescent signals were captured with a ProgRes MFCOOL camera (Jenoptik, Jena, Germany) at a magnification of 40X. For IF, image acquisition, deconvolution and Extended Depth of Focus on Z-serial optical sections were performed using NisElements AR 4.2 (Nikon, Minato, Tokyo, Japan). Images for each sample were taken in parallel using identical microscope settings. Quantifications of IF signals were performed using ImageJ software (NIH, Bethesda, MD, USA).

### 2.10. Live-Cell Imaging

HeLa cells cultured in DMEM without phenol red, with 10% FBS were seeded on 15μ-Slide 8 well (80826, ibiTreat, Ibidi), kept in a microscope stage incubator (Basic WJ, Okolab, Pozzuoli, Naples, Italy) at 37 °C and 5% CO_2_ and observed under an Eclipse Ti inverted microscope using a Plan Apo 40x objective (Nikon). DIC images were acquired every 3.5 min over a 28-h period by using a DS-Qi1Mc camera (Nikon) and the NIS-Elements AR 3.22 (Nikon).

### 2.11. Anti-HIPK2-S Rabbit Polyclonal Ab Production and Purification

Ab production and purification was performed by Thermo Fisher Scientific. Rabbits were immunized with the amide-conjugated peptide DSLVPGNLGPGQGRNL. Since there are no presumptive immunogenic peptides in the HIPK2-S specific protein sequence, we selected a peptide in which 5 out of 16 aa overlap to the region common to HIPK2-FL and HIPK2-S isoforms. To enrich the Ab specifically recognizing HIPK2-S protein, serum production was followed by a standard purification protocol with positive selection with a HIPK2-S specific peptide as immobilized antigen (GNLGPGQGRNLSLES) and negative adsorption with a HIPK2-FL peptide (TQASEVLVECDSLVP).

### 2.12. Statistics

The experiments were repeated three to five times and the results obtained presented as mean ± standard deviation (SD). *P* values were derived from unpaired two-tailed *t*-tests using GraphPad Prism software (GraphPad Software, Inc., San Diego, CA, USA), unless otherwise specified. *P*-values < 0.05 were considered significant.

## 3. Results

### 3.1. Detection in Human Cells of the Predicted HIPK2 Alternative Splice Variant with Intron 13 Retention

In silico interrogation of UCSC and NCBI RefSeq collections and human ESTs datasets was performed by using human HIPK2 (NM_022740) as a query sequence. Together with the already described Δe8 variant (NM_001113239), we obtained various RNA transcripts spreading into intron 13, while no other HIPK2 exon shows ESTs protruding from exon boundaries (Figure S1a). A fraction of these intronic ESTs are polyadenylated, suggesting that they originate from functional RNAs rather than being transcriptional artefacts (Figure S1b). This hypothesis is supported by two pieces of additional in silico data: (i) the finding of the same polyadenylation site in the PolyA-Seq collection obtained by digital gene expression and global mapping of polyadenylation sites [54] and (ii) the existence of transcripts spreading into intron 13 in publicly available RNAseq data obtained from nine different human cell lines (Figure S1c).

**Figure 1:**
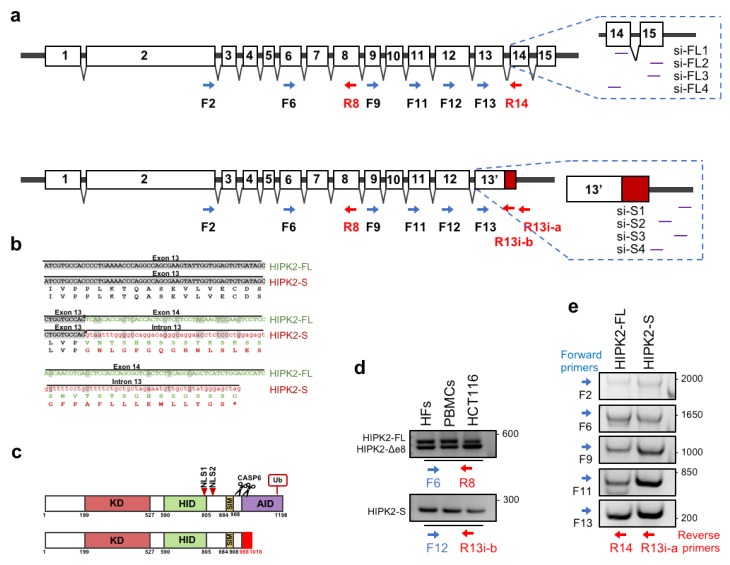
Identification of a new alternative splice variant of HIPK2. (**a**) Schematic representation of the exon composition of HIPK2-FL (upper) and HIPK2-S (lower) isoforms. In red is depicted the HIPK2-S specific sequence that is translated. In the enlarged boxes are indicated the annealing localizations of specific forward (F) and reverse (R) PCR primers and si-S and si-FL siRNAs. (**b**) Sequence alignment and relative translation of HIPK2-FL and HIPK2-S mRNA sequences. In light gray is highlighted the sequence common to both isoforms while the HIPK2-FL and HIPK2-S specific sequences are indicated in green and red, respectively. (**c**) Schematic illustration of HIPK2-FL and HIPK2-S protein isoforms specifying protein domain organization. KD: Kinase Domain; HID: Homeodomain Interacting Domain; NLS: Nuclear Localization Signal; SIM: Sumo Interacting Motif; CASP6: Caspase 6 cleavage sites; AID: Autoinhibitory Domain; Ub: Ubiquitination site. (**d**) RT-PCR using the indicated forward and reverse primers showed the presence of HIPK2-FL and HIPK2-∆e8 (upper panel) and HIPK2-S (lower panel) in different human cell types. (**e**) Different RT-PCRs were performed using the indicated combination of forward and reverse primers. In particular, the reverse primer R14 anneals on exon14 and amplifies HIPK2-FL (left lane) and the reverse primer R13i anneals on intron13 and amplifies HIPK2-S (right lane). As forward primers were used oligonucleotides annealing to exons from 13 (lower panel) to 2 (upper panel).

At the sequence level, this alternative splicing with intron 13 retention leads to the loss of exons 14 and 15 and to the gain of 89 bp, followed by a new stop codon and a new untranslated terminal region (Figure 1a). At the protein level, this alternative spliced RNA results in a short HIPK2 isoform (HIPK2-S) in which the C-terminal region of HIPK2-FL (aa 989–1198) is substituted with 30 new aa (Figure 1b), generating a protein of 1018 aa that lacks a large portion of the autoinhibitory domain (AID) and the ubiquitylation site (Figure 1c) [1,2,3]. An identical conceptual translated protein has been reported by Venter et al. [58].

Further analyses indicate that the intronic portion of HIPK2-S is well conserved (>90%) among the higher evolved primate families, while nucleotide similarity rapidly drops as one descends the phylogenetic tree (Figure S2a). In parallel, the alignment of in silico translated protein sequences shows that only primates closer to humans express a comparable HIPK2-S isoform (Figure S2b).

Next, we began evaluating whether HIPK2-S mRNA can be detected in human cells. By RT-PCR with primer pair specific for intron 13 (Figure 1a, Table 1), we amplified HIPK2-S mRNA in human cells that express HIPK2-FL and HIPK2-∆e8 isoforms, such as immortalized human fibroblasts (HFs), freshly isolated peripheral blood mononuclear cells (PBMCs), and HCT116 colon cancer cells (Figure 1d). These cells were selected to include primary, nontransformed, and tumor cells and to exclude cell line-specific behavior. To further characterize HIPK2-S mRNA, we performed sequential RT-PCR reactions using two fix reverse primers, the first annealing with intron 13 to detect HIPK2-S, and the second annealing with exon 14 to detect HIPK2-FL. In combination with the above reverse primers, we used a combination of forward primers moving back along the HIPK2 mRNA until exon 2 (Figure 1a). As shown in Figure 1e, fragments of comparable sizes were amplified from both HIPK2-S and HIPK2-FL isoforms, suggesting that HIPK2-S contains all the exons upstream of intron 13, including exon 2, which is translated into the kinase domain (Figure 1c).

### 3.2. Tissue and Tumor Expression of HIPK2-FL and HIPK2-S Isoforms

**Figure 2:**
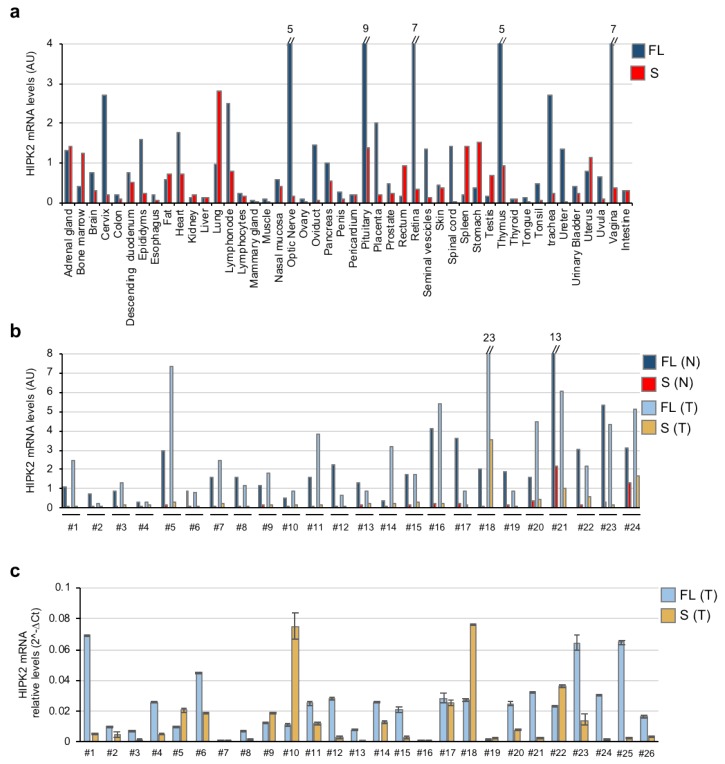
HIPK2-FL and HIPK2-S expression in normal and tumor tissues. (**a**,**b**) qPCR analysis of the expression levels of HIPK2-FL and HIPK2-S in different human, normal, tissue samples (**a**) and in a panel of human colon cancers and matched normal tissue (**b**). Values are shown as 2^-Ct multiplied by an arbitrary factor. (**c**) The expression levels of HIPK2-FL and -S were analyzed in different PDAC biopsies. *GAPDH* gene expression was used for data normalization. Data show a non-normal distribution by Shapiro‒Wilk normality test (*p* < 0.01 for FL and *p* < 0.0001 for S).

To determine whether and at what level the HIPK2-S isoform is expressed in normal tissues compared with HIPK2-FL, a panel of commercially available first-strand cDNAs from human tissues was analyzed by qPCR with isoform-specific primers (Table 1). The presence of RNAs of both isoforms is detectable in all tissues analyzed, usually at low levels. We found higher expression of HIPK2-FL compared to HIPK2-S in the optic nerve, pituitary gland, retina, thymus, cervix, vagina, and placenta. In contrast, the tissues in which the HIPK2-S expression is higher than the HIPK2-FL are the bone marrow, spleen, lung, stomach, rectum, and testis (Figure 2a).

Next, we analyzed the expression of the two HIPK2 isoforms in a commercially available panel of first-strand cDNAs prepared from 24 human colon cancers and matched nontumor tissue and in a cohort of 26 mRNAs from randomly selected pancreatic ductal adenocarcinomas (PDAC) banked at the Regina Elena Cancer Institute. We observed a large variation in the expression levels of the two HIPK2 isoforms in the different tumor biopsies and in normal versus tumor tissues (Figure 2b,c), indicating tumor-associated variability.

Therefore, similar to the HIPK2-FL isoform, the HIPK2-S variant is ubiquitously expressed in normal tissues, although at different levels in different tissues. Moreover, alterations of the relative expression of the two isoforms can be observed in human colon and pancreatic cancers, though, at this point, we cannot assign any specific predictive and/or prognostic value to these data.

### 3.3. Cloning and Expression of the HIPK2-S Isoform

**Figure 3:**
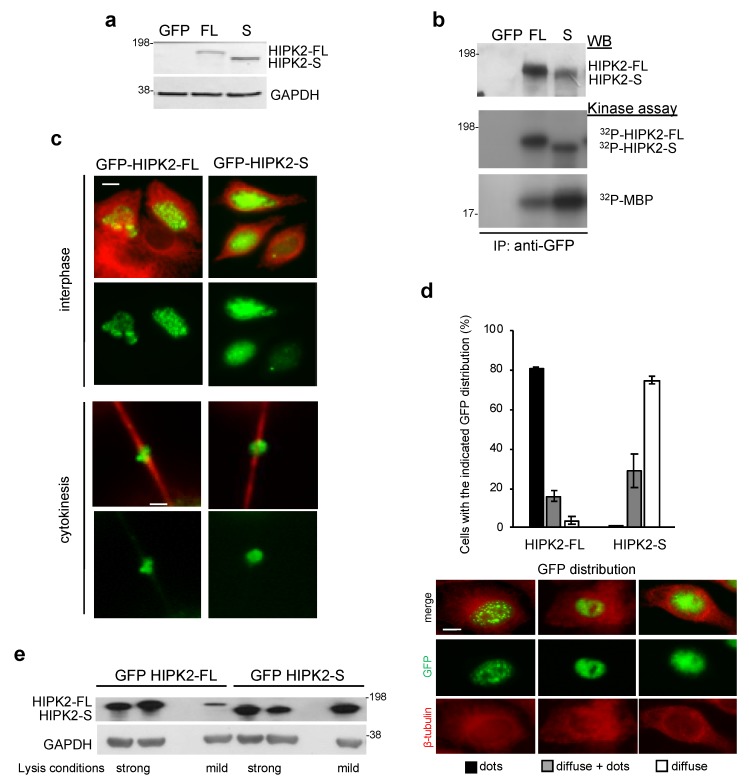
Distribution and kinase activity of GFP-HIPK2-S compared to GFP-HIPK2-FL. (**a**) U2OS cells were transfected with the GFP empty vector (GFP) or with the vectors expressing GFP-tagged HIPK2-FL (FL) or HIPK2-S (S) and analyzed 24 h post-transfection. Representative WB of three different transfections with anti-GFP Ab is shown. Molecular weight markers are reported here and in the following figures in KDa. (**b**) In vitro kinase assays were performed by using Myelin Basic Protein (MBP) as a substrate in combination with GFP, GFP-HIPK2-FL (FL), and GFP-HIPK2-S (S) expressed and immunoprecipitated in U2OS. Proteins were separated by SDS-PAGE and radioactivity detected by autoradiography. WB (upper panel) was performed as loading control. (**c**) Representative images of proliferating HeLa cells transfected as the U2OS cells in (**a**) and analyzed for GFP autofluorescence (green) and by IF with anti-β-tubulin Ab (β-Tub, red) to mark cytoplasm in interphase and midbodies in cytokinesis, respectively. Bars indicate 5 μm (left panels) and 1 μm (for midbody magnification, right panels). (**d**) HeLa cells were transfected and analyzed as in (**c**). The indicated types of GFP distribution shown in the left panels were quantified and reported as mean ± standard deviation (SD). Three independent experiments were performed and total 150 cells per condition were analyzed. Bar indicates 10 μm. (**e**) U2OS cells were transfected as in (**a**) and 48 h after transfection harvested and lysed with hot Laemmli buffer (strong) or SDS/NP40 lysis buffer (mild) and analyzed by WB with anti-GFP Ab. One representative WB of two independent experiments is reported. GAPDH was used as the loading control.

To determine whether HIPK2-S mRNA leads to protein expression and to characterize HIPK2-S protein activity and subcellular localization compared with HIPK2-FL, we cloned the synthetically generated cDNAs of the two variants into a mammalian expression vector in frame with a GFP-tag and verified their expression by WB upon transfection in human cells (Figure 3a). In vitro kinase assays performed on the immunoprecipitated proteins with an anti-GFP antibody (Ab) showed that, like the FL isoform, HIPK2-S is enzymatically active (Figure 3b). Evaluation of GFP-HIPK2-FL and GFP-HIPK2-S subcellular localization by tag-autofluorescence showed that both isoforms can be detected in the nucleus of interphase cells and at the ICB of dividing cells upon transfection in different cell types (e.g., HeLa, HFs, U2OS, HCT116) (Figure 3c; data not shown). However, deeper assessment showed that GFP-HIPK2-S has a more diffuse and less dotted nuclear distribution than the typical HIPK2-FL localization (Figure 3d and Figure S3). This difference was confirmed by WB analyses of whole cell lysates obtained with strong and mild extraction buffers, showing that only HIPK2-S can be easily extracted in mild conditions (Figure 3e).

### 3.4. HIPK2-S Isoform Is Dispensable for DNA Damage Response

**Figure 4:**
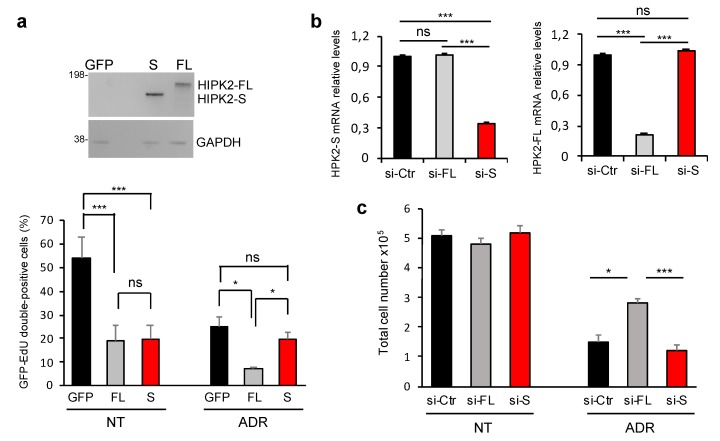
HIPK2-S isoform is dispensable for DNA damage response. (**a**) U2OS cells were transfected with expression vectors for the indicated GFP-tagged HIPK2 isoforms and analyzed by WB as in Figure 3a. Cells were maintained in nontreated (NT) condition or treated with 0.6 μM ADR for 48 h (ADR). Inhibition of cell proliferation was assessed by EdU-incorporation and subsequent fluorescence. The percentage of EdU-positive cells was separately measured in at least 100 GFP-positive and 100 GFP-negative cells per dish. Transfected GFP-positive cells and nontransfected GFP-negative cells belong to the same dishes. Data represent the mean ± SD of three independent experiments. * *p* < 0.05; *** *p* < 0.001; ns *p* > 0.05. (**b**) U2OS cells were transfected with siRNAs specific for HIPK2-FL (si-FL 1 and 2, see Table 2) and HIPK2-S (si-S 1 and 2, see Table 2) or the universal negative control (si-Ctr) and analyzed 48 h post-transfection by qPCR to verify isoform-specific RNA interference. Histograms show data from one representative experiment out of four performed with two different siRNA mix (si-S1/si-S2 and si-FL1/si-FL2 or si-S3/si-S4 and si-FL3/si-FL4) and producing comparable results. (**c**) U2OS cells were transfected as in (**b**) and 48 h post-transfection cells were treated (ADR) or not (NT) with Adriamycin. Cell number was evaluated with an automated cell counter. Data represent the mean ± SD of three independent experiments. * *p* < 0.05; *** *p* < 0.001.

To investigate whether HIPK2-S is linked to specific HIPK2-mediated functions, we transfected the two GFP-tagged isoforms or the GFP-empty vector at the same molar ratio and replica dishes of the three transfected populations were treated or not with Adriamycin (ADR). Proliferation rates were measured by single-cell analyses of EdU incorporation in GFP-positive, transfected cells, and in GFP-negative, nontransfected cells, in the same dish (Figure 4a and Figure S4). Similar inhibition of EdU positivity was observed in the GFP-positive cells in the untreated samples indicating that HIPK2-S overexpression per se reduces proliferation rate as well as HIPK2-FL (Figure 4a, NT-columns). However, when genotoxic stress was induced by ADR treatment, FL-expressing cells showed the expected intensification of growth arrest while S-expressing cells were not further affected (Figure 4a, ADR-columns) and behaved similarly to the GFP-negative, control cells of same dishes (Figure 4a and Figure S4).

To verify whether HIPK2-S is excluded from DNA damage response as suggested by the above experiments, we selectively inhibited HIPK2-FL and HIPK2-S isoforms with specific short interfering RNAs (siRNAs) (Figure 1a, Table 2).

Because of the sequence employed, depletion of the FL isoform (si-FL) includes the HIPK2-Δe8 isoform, which was shown to have a functional activity similar to HIPK2-FL’s [52,53]. In contrast, the HIPK2-S siRNAs (si-S) were specific for this isoform, as confirmed by specific qPCRs (Figure 4b). When treated with ADR, si-FL cells, as expected, show increased survival compared to the control cells (si-Ctr). In contrast, si-S cells do not modify their response to ADR treatment, suggesting that HIPK2-S is dispensable for this function (Figure 4c).

### 3.5. HIPK2-S Isoform Is Required for Faithful Cytokinesis

**Figure 5:**
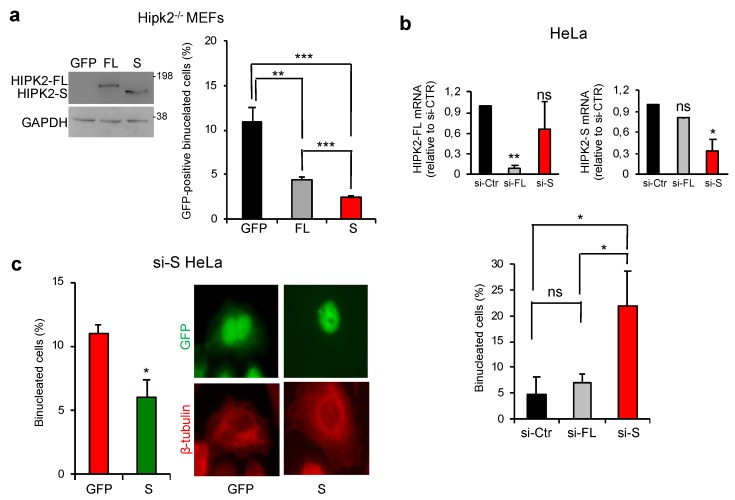
HIPK2-S but not HIPK2-FL is required for successful cytokinesis. (**a**) MEFs from Hipk2^-/-^ mice were transfected with the GFP empty vector (GFP) the vectors expressing GFP-tagged HIPK2-FL (FL) or HIPK2-S (S) and analyzed 48 h post-transfection for protein expression by WB with anti-GFP Ab. MEFs from the same transfection were maintained in culture for additional 24 h and analyzed for the presence of binucleated cells by GFP autofluorescence, and IF with anti-β-tubulin Ab (red) to mark cytoplasm and DAPI to mark nuclei. The percentage of binucleation was measured in the GFP-positive cells in the three transfected populations (at least total 150 cells per condition were analyzed). Data represent the mean ± SD of three independent experiments. ** *P* < 0.01; *** *P* < 0.001. (**b**) HeLa cells were transfected with the indicated stealth siRNAs (see Table S2) and analyzed 72 h post-transfection by qPCR to verify RNA interference and 96 h post-transfection by IF after staining with DAPI and anti-β-tubulin Ab to visualize cytoplasm and score binucleated cells (at least 300 cells in total per condition were analyzed) as shown in (**a**). Values are mean ± SD of three independent experiments. * *p* < 0.05 and ** *p* < 0.001, t-test relative to si-Ctr (in left panels). (**c**) HIPK2-S depleted cells (si-S) were obtained as in (**b**), transfected with GFP or GFP-HIPK2-S 48 h post-siRNA transfection and analyzed by IF as in (**b**). Note that in si-S cells the expression of GFP-HIPK2-S is not inhibited by RNAi because the employed siRNAs recognize sequences that are not present in the GFP-HIPK2-S expressing vector (i.e., downstream HIPK2-S stop codon; Figure 1a). The percentage of binucleated cells relative to the GFP-positive cells is reported as mean ± SD of two independent experiments, in which at least total 200 cells per condition were analyzed. * *p* < 0.05. Representative images are reported.

Since exogenous GFP-HIPK2-S localizes at the ICB, we asked whether the short isoform plays a role in cytokinesis. We first transfected GFP-HIPK2-FL and GFP-HIPK2-S in low passage mouse embryo fibroblasts (MEFs) from Hipk2^−/−^ mice. Such cells display an increased number of binucleated cells compared to the Hipk2^+/+^ counterpart due to cytokinesis failure [42]. To avoid HIPK2-induced growth arrest, low amount of plasmid DNA was transfected. As expected from previous observation, GFP-HIPK2-FL reduced the number of binucleated cells (Figure 5a, black and grey columns). However, comparable amount of GFP-HIPK2-S significantly further reduced binucleation (Figure 5a, red column). Next, selective inhibition of the two isoforms were performed in HeLa cells and the accumulation of binucleated cells was evaluated 96 h after transfection of isoform-specific siRNAs (Figure 5b). We observed that si-FL does not cause binucleation while si-S significantly induces accumulation of binucleated cells (Figure 5b). Comparable results were obtained in HFs and U2OS cells and with an independent set of siRNAs, such as chemical modified stealth siRNAs with different annealing sequences (Figure S5, Table 2), excluding cell-specific and sequence-specific behaviors. Of note, cell division was rescued by expressing GFP-HIPK2-S in the si-S cells further excluding off-target effects (Figure 5c).

**Figure 6:**
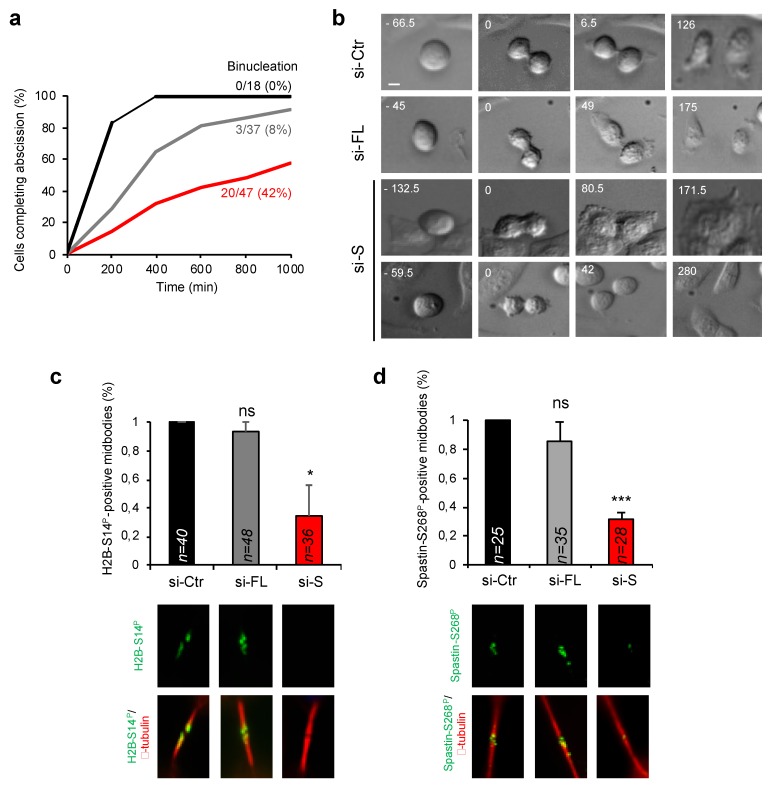
HIPK2-S but not HIPK2-FL is required for successful cytokinesis. (**a**) HeLa cells were transfected as in Figure 5b and analyzed by time lapse imaging 70 h post-transfection. The abscission time, i.e., from cleavage furrow ingression to abscission, was determined and the cumulative percentage of the analyzed cells (si-Ctr, *n* = 18; si-FL, *n* = 37; si-S, *n* = 47) from three independent transfections is plotted as a function of time. The percentage of cells that fail cytokinesis and undergo binucleation is also reported. (**b**) Representative stills from time-lapse imaging of cells recorded in (**a**). The time from the beginning of the round-up is shown in h:min. Scale bar is 10 μm. The relative videos are Videos S1‒S4. (**c**,**d**) HeLa cells were transfected as in (**a**) and analyzed 96 h post-transfection by IF after staining with anti-phospho-H2B-S14 or anti-phospho-spastin-S268 Abs in combination with anti-β-tubulin to mark midbody. Data are reported as mean ± SD of three independent experiments * *p* < 0.05; *** *p* < 0.0001. Representative images of midbody magnification are reported below each column, while the numbers of analyzed midbodies are reported inside the relative columns.

To further evaluate the cytokinesis contribution of HIPK2-S, we performed time-lapse live-cell imaging of asynchronous HeLa cells after selective depletion of HIPK2-FL and HIPK2-S isoforms. Analysis of the abscission time (i.e., the timing from cleavage furrow ingression to physical separation of the two daughter cells) showed that si-Ctr cells end abscission in less than 400 min, while only 60% of the si-S cells can complete abscission and in a significantly longer time. In contrast, si-FL cells behave more similarly to si-Ctr cells with only three out of 37 cells failing division and an abscission time over 600 min in 19% of the cells (si-Ctr, *n* = 18; si-FL *n* = 37; si-S, *n* = 47; Figure 6a,b and Videos S1‒S4).

Finally, these same isoform-depleted HeLa cells were analyzed for the HIPK2-mediated phosphorylation at the ICBs of the two known cytokinesis targets of HIPK2, histone H2B and Spastin [45,46]. IF analyses were performed with anti-phospho-H2B-Ser14 and anti-phospho-Spastin-Ser268 Abs that recognize the HIPK2-phosphorylated sites on H2B and Spastin, respectively. Significant inhibition of H2B-Ser14 and Spastin-Ser268 phosphorylation at the ICB was detected in si-S cells while si-FL cells behaved similarly to si-Ctr (Figure 6c,d).

Taken together, these results indicate that the cytokinesis failure previously observed in pan-HIPK2-deficient cells is due to the absence of the HIPK2-S isoform rather than the HIPK2-FL protein.

### 3.6. Endogenous HIPK2-S Is Enriched in Telophase and Localizes at the ICB of Dividing Cells

**Figure 7:**
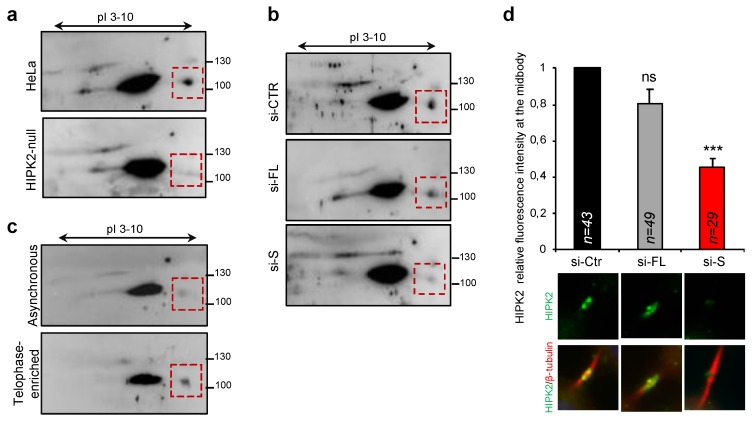
(**a**,**b**) HeLa cells and their indicated derivatives were lysed and total cell extracts separated onto parallel 2D SDS-PAGE. The HeLa derivative are: (i) HIPK2-null, obtained by CRISPR/Cas9; (ii) si-CTR, parental HeLa cells transfected with the universal negative siRNA control; (iii) si-FL, parental HeLa cells transfected with si-FL1/si-FL2 siRNAs; and (iv) si-S, parental HeLa cells transfected with si-S1/si-S2 siRNAs. WB were performed with anti-HIPK2 rabbit polyclonal Ab by L.M. Schmitz. Red square boxes highlight the HIPK2-S isoform. (**c**) Parental HeLa cells were maintained in proliferating, asynchronous condition or synchronized and enriched in telophase and analyzed as in (**a**,**b**). (**d**) Parental HeLa cells were transfected as in Figure 5b and analyzed 96 h post-transfection by IF after staining with anti-HIPK2 (pan-HIPK2, Ab946) in combination with anti-β-tubulin Ab to mark midbody. Data are reported as mean ± standard error of mean (SEM) of three independent experiments *** *p* < 0.0001. Quantification of IF signals was performed by measuring the mean of pixel intensity at midbody corrected for external background. Representative images are reported below each column.

To determine whether endogenous HIPK2-S isoform can be detected in standard cell culture conditions, we first tried to produce and validate an anti-HIPK2-S rabbit polyclonal Ab. As reported for the majority of the anti-HIPK2 Abs, our anti-HIPK2-S Ab specifically recognizes the exogenous protein, but not the endogenous one (Figure S6). To overcome this limitation, total cell extracts from parental HeLa cells and their HIPK2-null derivatives obtained by CRISPR/Cas9 [59] were analyzed by 2D gels and WB with an anti-HIPK2 rabbit polyclonal Ab raised with a protein region present also in the HIPK2-S isoform [23]. A spot of the expected molecular weight (MW) was detectable in the parental (HIPK2-WT) cells, but not in the HIPK2-null derivatives (Figure 7a). This spot has a similar MW to a very abundant protein that cross-reacts with the anti-HIPK2 Ab, thus, we could only perform 2D WB. To confirm that the 100 KDa spot is indeed the HIPK2-S isoform, the parental HIPK2-WT HeLa cells were transfected with control, HIPK2-FL-, and HIPK2-S-specific siRNAs as above and analyzed by 2D WB. Strong reduction of the same 100 KDa spot was observed only in the si-S cells, confirming its specificity (Figure 7b).

Because of HIPK2-S’s role in cytokinesis, we next assessed whether this isoform can be enriched in telophase and be specifically detected at the ICB. Asynchronous, proliferating HeLa cells and telophase-enriched cells—obtained by cell synchronization, mitotic shake-off, and replating for 90/100 min—were lysed and analyzed by 2D WB as above. A clear enrichment of the 100 KDa spot was observed in telophase-enriched cells (Figure 7c). To directly detect endogenous HIPK2 at the ICB, IF analyses were performed with the anti-HIPK2 Ab946 that recognizes HIPK2 at the ICB [42]. Qualitative and quantitative evaluation of ICB immunostaining shows a significant inhibition of HIPK2 detection only in the si-S cells while the FL-depleted cells are similar to the controls (Figure 7d). Therefore, the HIPK2-S isoform is expressed in standard culture conditions and localizes at the ICB for faithful cell division.

## 4. Discussion

In this study, we investigated whether alternative splicing isoforms of HIPK2 can help to understand the divergent functions of HIPK2 and to explain the apparently contradicting data reported thus far. By a combination of in silico analysis and in vitro experiments, here we unravel and characterize a new alternative splice variant of HIPK2 with intron 13 retention, namely the HIPK2-S isoform. Compared to HIPK2-FL, the HIPK2-S variant lacks a large portion of the AID at the C-terminal region (aa 989–1198) and gains 30 new aa generating a protein of 1018 aa.

By employing two different pairs of siRNAs annealing to isoform-specific regions, we selectively inhibited either the new shorter isoform HIPK2-S or the two functionally related longest isoforms HIPK2-FL and HIPK2-∆e8. This allowed us to discriminate between the cytokinesis function played by the endogenous HIPK2-S isoform and the DNA-damage response function played by the endogenous HIPK2-FL.

The mechanism underlying the distinct HIPK2 activities is presently unknown. Overexpressed GFP-tagged isoforms can be detected in both the nucleus and the ICB. In the nucleus, the HIPK2-FL protein exhibits the well described nuclear speckle distribution, while the HIPK2-S isoform is more diffused and dispersed in the entire nucleoplasm. Whether this difference results in a mild/absent activity in the regulation of gene expression of the HIPK2-S isoform has to be defined. Based on the results we obtained by isoform-specific depletion, we focused our attention on ICB localization and activity. Indeed, though both overexpressed GFP-tagged isoforms can localize at the ICB and inhibit binucleation in Hipk2^-/-^ MEFs, HIPK2-S does so more efficiently than HIPK2-FL. Notably, IF analyses of endogenous HIPK2 proteins obtained with an anti-panHIPK2 Ab showed a strong reduction of the HIPK2 signal at the ICB only in HIPK2-S depleted cells, suggesting that, in non-overexpressing conditions, HIPK2-S is the only isoform that localizes at the ICB.

At this point, whether the ICB localization depends on the missing AID or the gained new aa is unclear. An anti-HIPK2-S-specific Ab would definitely have improved our ability to study this aspect, while the use of deletion mutants would not help since it would fall into the caveat of exogenous overexpressed proteins [15]. Unfortunately, as frequently observed with anti-HIPK2 Abs, they detect exogenous but not endogenous proteins [15]. Interestingly, the missing AID region mainly overlaps with the portion that is cleaved at aa 916 and 977 by caspase in the HIPK2-FL isoform [60]. Such caspase-cleaved form of HIPK2-FL has been shown to lose the ability to bind a multiprotein complex containing histone deacetylases, alleviating its repressive function on gene expression during muscle differentiation. These data argue for a transcription-related function of the AID-truncated HIPK2 form [60,61] rather than a transcription-independent activity in cytokinesis.

Though no direct experiment has been performed yet, based on the data reported on the caspase-cleaved HIPK2-FL, it can be hypothesized that the newly gained 30 aa might be relevant for ICB localization and related activity. The sequencing of the human genome [58] identified a HIPK2 isoform named isoform CRA_d (GenBank accession code EAW83928), whose conceptual translation is identical to that of HIPK2-S. The HIPK2-CRA_d name was based on the presence of a putative C-terminal CT11-RanBPM (CRA) domain, a protein‒protein interaction domain originally described in the Ran-binding protein, RanBPM [57]. The CRA domain of RanBPM is required for the interaction with fragile X mental retardation protein, FMRP. By analogy, the CRA domain of HIPK2-S might be required for the complex network of interactions among the microtubule modulators and their regulators during ICB assembly.

Alternative splicing is a major diversification mechanism to increase complexity in mRNA and proteins [48,49]. Several diseases, including cancers, have been associated with the dysregulation of alternative splicing. By measuring the mRNA levels of HIPK2-FL and HIPK2-S isoforms in different tumor biopsies and in normal versus tumor tissues, we observed alterations in the relative expression of the two isoforms. This observation opens up the possibility of testing whether tumor-specific isoform pattern(s) might contribute to tumorigenesis and might also be used as potential biomarkers.

## Figures and Tables

**Table 1:** HIPK2 isoform specific primers.

**RT-PCR primers**		
F2	Forward	5′-CAGCCAGCCACGTCTCCAAGG-3′
F6	Forward	5′-GGCTGACCGGCGGGAGTT-3′
F9	Forward	5′-CCCATCGTCACTCAAGCCCCAG-3′
F11	Forward	5′-AGCAGCCAACCAGCACCACCT-3′
F12	Forward	5′-AGGAGGAACAGAAACACGCC-3′
F13	Forward	5′-CCTGAAAACCCAGGCCAGCGA-3′
R14	Reverse	5′-CGCTGCTGCCGGTAGGTGATG-3′
R13i-a	Reverse	5′-GCCTGCAGTCACTGGGCACTT-3′
R13i-b	Reverse	5′-CCTGTCCTGGCCCCAAAT-3′
R8	Reverse	5′-GGTCAGGCCGGGCACAAATCT-3′
**qPCR primers**		
HIPK2-S	Forward	5’-AGGAGGAACAGAAACACGCC-3’
HIPK2-S	Reverse	5’-CCTGTCCTGGCCCCAAAT-3’
HIPK2-FL	Forward	5’-AGGAAGAGTAAGCAGCACCAG-3’
HIPK2-FL	Reverse	5’-TGCTGATGGTGATGACACTGA-3’

**Table 2:** HIPK2 isoform specific siRNAs.

si-S1	5′-AUCCUGUAAUCAAUACCUCTT-3′	siRNA/MWG
si-S2	5′-GGACAUUGUAUAAGCAGCGTT-3′	siRNA/MWG
si-FL1	5′-UCACUCUUCAGGGAGCUCAUCUGGA-3′	siRNA/MWG
siFL2	5′-CCAAGGUCAACCAGUACCCUUACAU-3′	siRNA/MWG
GL2^§^	5′-CGUACGCGGAAUACUUCGAUU-3′	siRNA/MWG
si-S3	5′-UCUUUAAUCCUGUAAUCAAUACCUC-3′	stealth siRNA/Invitrogen
si-S4	5′-GCUUCUGCUGAACAGCACAUUGUAU-3′	stealth siRNA/Invitrogen
si-FL3	5′-UCACUCUUCAGGGAGCUCAUCUGGA-3′	stealth siRNA/Invitrogen
si-FL4	5′-CCAAGGUCAACCAGUACCCUUACAU-3′	stealth siRNA/Invitrogen
UNC*	Negative Medium GC Duplexes	stealth siRNA/Invitrogen

^§^ siRNA targeting the luciferase gene used as control. * Universal negative control stealth siRNA.

## Supplementary Materials

The following are available online at https://www.mdpi.com/2073-4409/9/2/484/s1, Figure S1: In silico analyses of HIPK2 isoforms, Figure S2: Phylogenesis of the HIPK2-S isoform, Figure S3: HIPK2-S shows a more diffuse nuclear staining than HIPK2-FL, Figure S4: HIPK2 isoforms in DNA damage response, Figure S5: HIPK2-S is required for successful cytokinesis in normal and tumor cells, Figure S6: Anti-HIPK2-S Ab production and validation, Video S1: Time-lapse microscopy of si-Ctr HeLa cells related to Figure 5, Video S2: Time-lapse microscopy of si-FL HeLa cells related to Figure 5, Video S3: Time-lapse microscopy of si-S HeLa cells undergoing binucleation, related to Figure 5 (si-S upper panels). Video S4: Time-lapse microscopy of si-S HeLa cells with long abscission time, related to Figure 5 (si-S lower panels).
