# Supplementary material for: An Alternative Splice Variant of HIPK2 with Intron Retention Contributes to Cytokinesis
Source: Cells. 2020 Feb 20;9(2):484. doi: 10.3390/cells9020484 (PMC7072727; doi:10.3390/cells9020484)
Supplement: Supplementary file 1 [file cells-09-00484-s001.zip › VG Supplementary Files/Gatti et al Supplementary Materials.pdf]

An alternative splice variant of HIPK2 with intron retention contributes to cytokinesis

Veronica Gatti, Manuela Ferrara, Ilaria Virdia, Silvia Matteoni, Laura Monteonofrio, Simona Di Martino, Maria Grazia Diodoro, Giuliana Di Rocco, Cinzia Rinaldo, Silvia Soddu

Supplementary Data

Table of Contents

1) Supplementary Figure S1

2) Supplementary Figure S2

3) Supplementary Figure S3

4) Supplementary Figure S4

5) Supplementary Figure S5

6) Supplementary Figure S6

Supplementary Figure S1

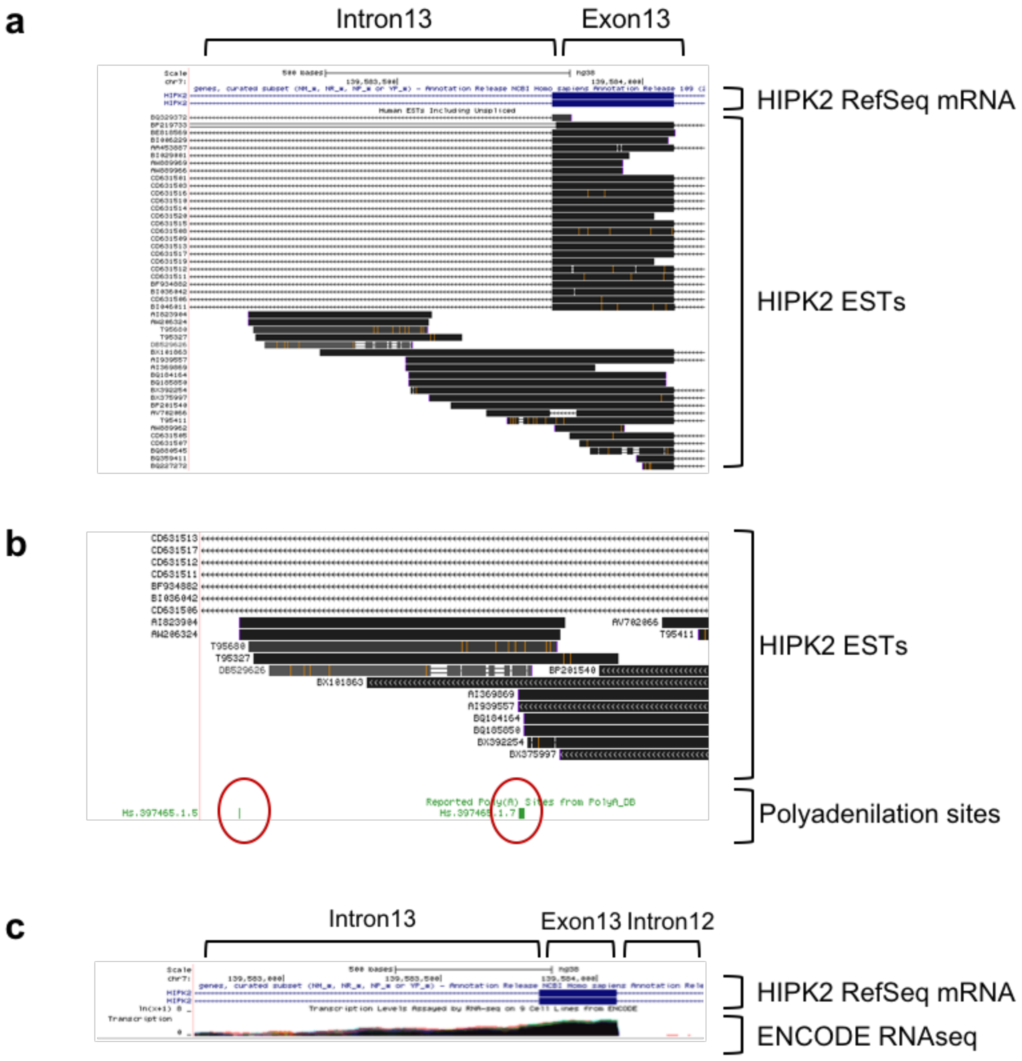

Figure S1. (related to Figure 1) In silico analyses of HIPK2 isoforms. (**a**) Screenshot taken from UCSC Genome Browser (https://genome-euro.ucsc.edu) representing annotated RefSeq sequences and expressed sequence tags (ESTs) including both spliced and unspliced. (**b**) Screenshot from UCSC Genome Browser indicating reported polyadenylation sites form polyA_DB database (http://exon.umdnj.edu/polya_db/). (**c**). Screenshot taken from UCSC Genome Browser showing high-throughput sequencing of polyadenylated RNA (RNAseq) signals from nine different human cell lines by ENCODE project (https://genome-euro.ucsc.edu/cgi-bin/hgTrackUi?g=wgEncodeCaltechRnaSeq).

Supplementary Figure S2

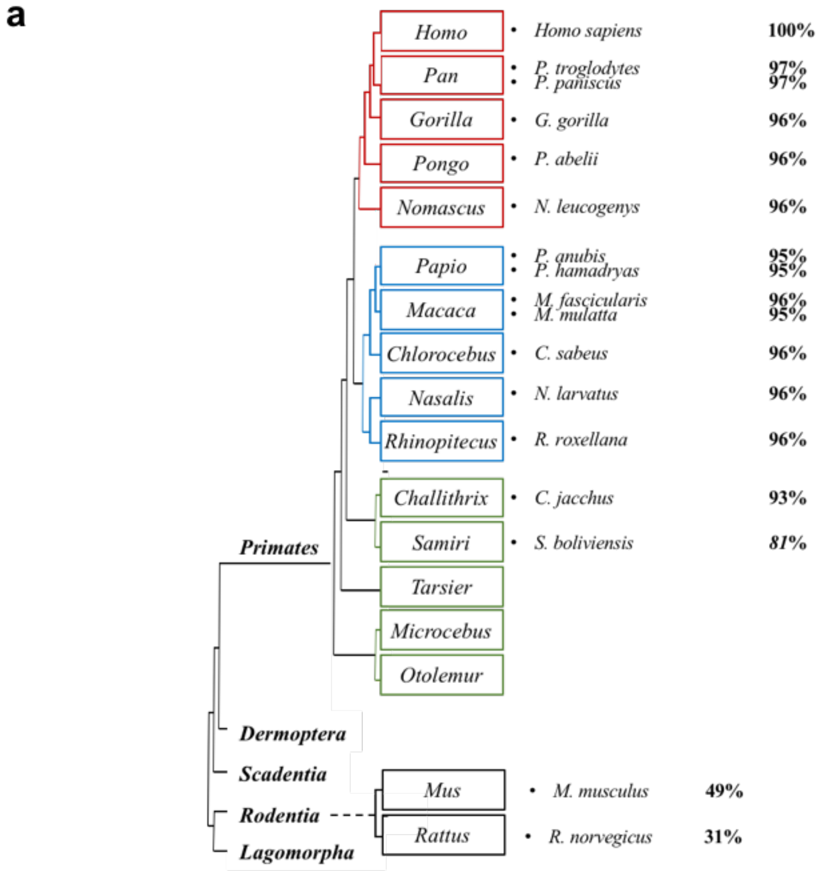

b

```
GNLGPGQGRNLSLESGFPAFLLLEMLLYGS*      Homo sapiens              100%
GNLGPGQDRNISLESGFPVFLLLEMLLYGS*      Pan troglodytes           90%
GNLGPGQDRNISLESGFPVFLLLEMLLYGS*      Pan paniscus              90%
GNLGPGQDRNISLESGFPVFLLLEMLLYGS*      Gorilla gorilla gorilla   90%
GNLGPGQDRNISLESGFPVFLLLEMLLYGS*      Pongo pygmaeus abelii     90%
GNLGPGQDRNISLESGFPVFLLLEMLLYGS*      Nomascus leucogenys       90%
GNLGPGQDRNISLESGFPVFLLLEMLLYGS*      Chlorocebus sabeus        90%
GNLGPGQDRNISLESGFPVFLLLEMLLYGS*      Macaca fascicularis       90%
GNLGQGQDRNISLESGFPVFLLLEMLLYGS*      Macaca mulatta            86,7%
GNLGPGQDRSISLESGFPVFLLLEMLLYGS*      Papio anubis              86,7%
GNLGPGQDRSISLESGFPVFLLLEMLLYGS*      Papio hamadryas           86,7%
GNLGPGQDRNISLESGFPVFLLLEMLLYGS*      Nasalis narvatus          90%
GNLGPGQDRNISLESGFPVFLLLEMLLYGS*      Rhinopitheous roxellana   90%
GNLGPGQDRNTSLESSFPAFLLLEMLLYGS*      Callithrix jacchus        86,7%
GNLGPGQDRNISLESSFPVFL----LLYGSWYNG   Saimiri boliviensis
GNLGPGQARTISLETGFPVFGC*              Tarsius syrichta

GNLGPQHSHAISLLFSFSAIRNVSTSETEHT*     Mus musculus
GNLGPR----ISLLFLFSAIRNVSTSETEHT*     Rattus norvegicus
```

Figure S2. (related to Figure 1) HIPK2-S sequence is conserved at nucleotide and aminoacidic level. (**a**) Phylogenetic tree illustrating sequence conservation among primates, indicated as percentage of nucleotide identity. Rat and mouse are also reported. (**b**) Predicted mRNA sequences were in silico translated and the resulting HIPK2-S specific peptide sequences were aligned. The percentage of aminoacid identity is indicated.

Supplementary Figure S3

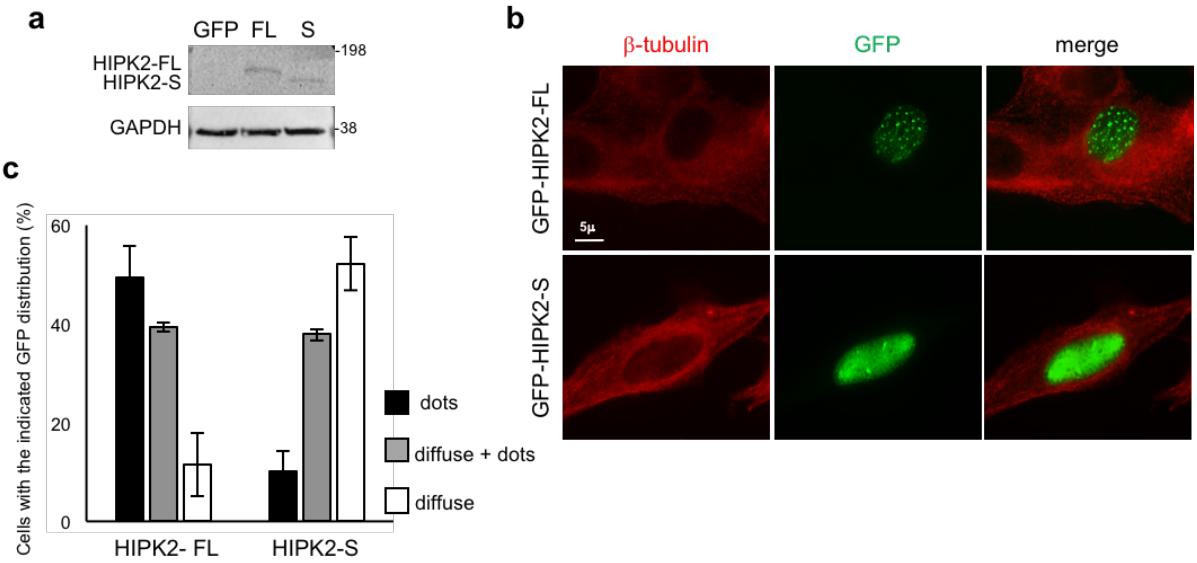

Figure S3. (related to Figure 3) HIPK2-S shows a more diffuse nuclear staining than HIPK2-FL also in immortalized human fibroblasts (HFs). (**a-c**) HFs were transfected with vectors expressing GFP-tagged HIPK2-FL or HIPK2-S and analyzed 24 h post-transfection by WB and IF after staining anti-β-tubulin to visualize cytoplasm (red). Representative WB and immunostainings are shown in **a** and **b**, respectively; bar, 5μM. Data quantification are reported in **c**, the values are mean ± SD from 2 independent experiments, in which total 130 cells per condition were analyzed.

Supplementary Figure S4

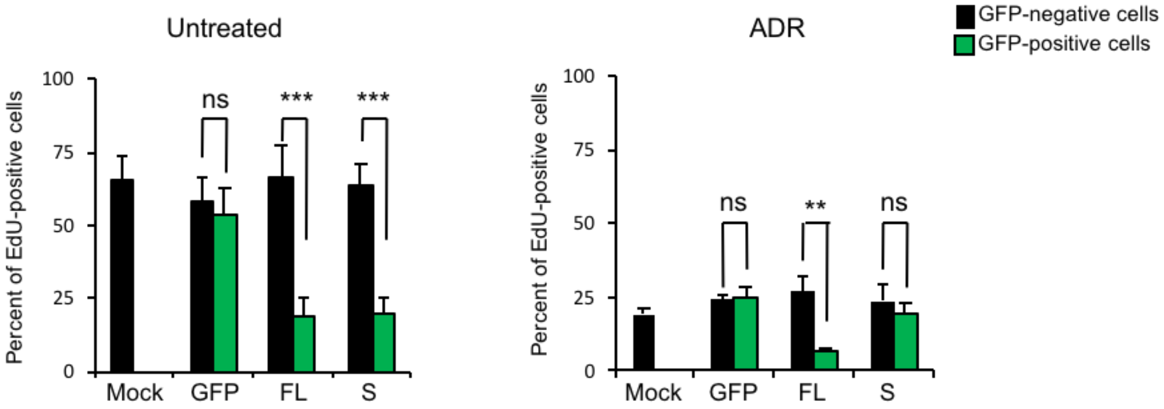

Figure S4. (related to Figure 4a) HIPK2 isoforms in DNA damage response. U2OS cells were transfected with expression vectors for the indicated GFP-tagged HIPK2 isoforms. Cells were maintained in non-treated (NT) condition or treated with 0.6 μM ADR for 48 h (ADR). Inhibition of cell proliferation was assessed by EdU-incorporation and subsequent fluorescence. The percentage of EdU-positive cells was measured in GFP-positive and GFP-negative cells. Transfected GFP-positive cells and non-transfected GFP-negative cells belong to the same dishes evaluated in Figure 4a. Data represent the mean ± SD of three independent experiments. ** $p<0.01$; *** $p<0.001$; ns $p>0.05$.

Supplementary Figure S5

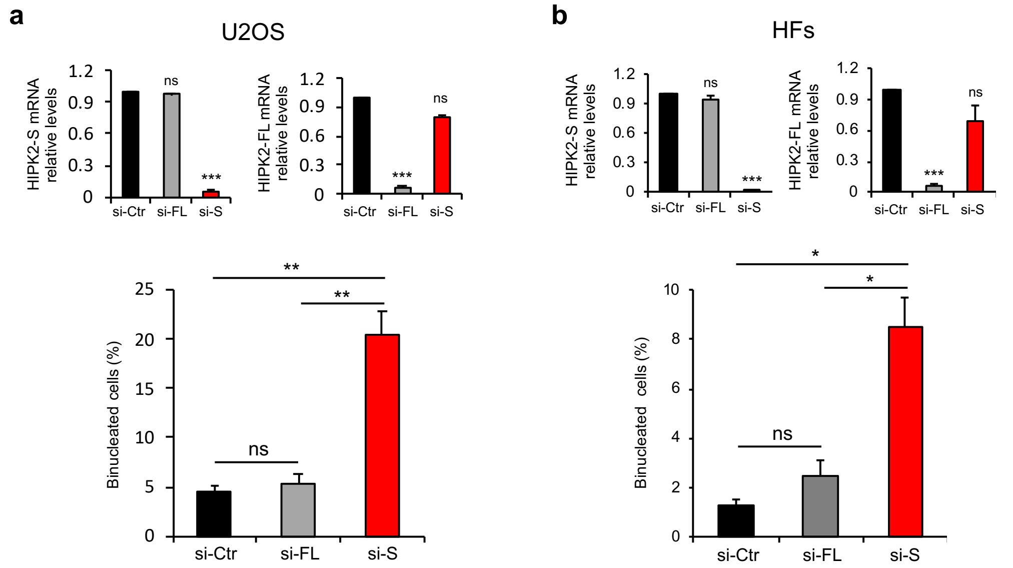

Figure S5. (related to Figure 5) HIPK2-S is required for successful cytokinesis in normal and tumor cells. (**a**) U2OS cells were transfected with the indicated siRNAs (numbers 1 and 2, see Table 2) and analyzed 72 h post-transfection by qPCR to verify RNA interference and by IF to score binucleated cells as described in Figure 5. At least 200 cells per condition were analyzed. Values are mean ± SD from 3 independent experiments ** p<0.001; *** p<0.0001. In the qPCR panels, t-test is relative to si-Ctr. (**b**) HFs were transfected with indicated stealth siRNAs (numbers 3 and 4, see Table 2) and analyzed 72 h post-transfection as in **a**. Values are mean ± SD from 2 independent experiments. * p<0.05; *** p<0.001. In the qPCR panels, t-test is relative to si-Ctr.

Supplementary Figure S6

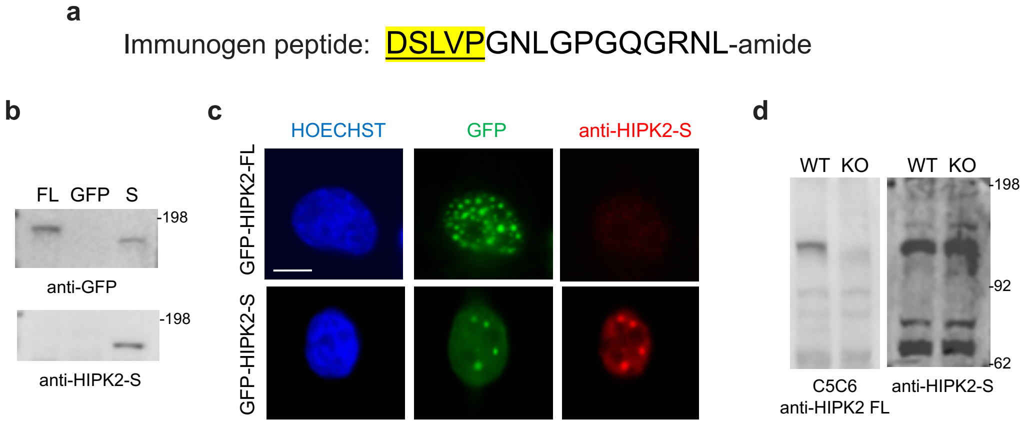

Figure S6. (related to Figure 6) Anti-HIPK2-S Ab production and validation. (**a**) Peptide sequence used for immunization is reported; the aminoacids in common with HIPK2-FL are underlined and highlighted in yellow. (**b**, **c**) HIPK2-null HeLa cells were transfected with vectors expressing GFP-tagged HIPK2-FL or HIPK2-S and analyzed 24 h post-transfection by WB and IF. Representative WB and immunostainings are shown. Bar, 5 μm. Data show that our anti-HIPK2-S Ab specifically recognizes exogenously expressed HIPK2-S isoform. (**d**) Total cell extracts from HIPK2 proficient (WT) and HIPK2-null (KO) HeLa cells were analyzed by WB using the C5C6 rat moAb (kindly provided by M.L. Schimtz), that recognizes only the HIPK2-FL isoform because was raised against the C-Ter region of HIPK2, and our anti-HIPK2-S Ab. No specific signal(s) was detected on the endogenous proteins with our anti-HIPK2-S Ab.
