# Supplementary material for: An Alternative Splice Variant of HIPK2 with Intron Retention Contributes to Cytokinesis
Source: Cells. 2020 Feb 20;9(2):484. doi: 10.3390/cells9020484 (PMC7072727; doi:10.3390/cells9020484)
Supplement: Supplementary file 1 [file cells-09-00484-s001.zip › VG Supplementary Files/WB row data.pptx]

## Slide 1
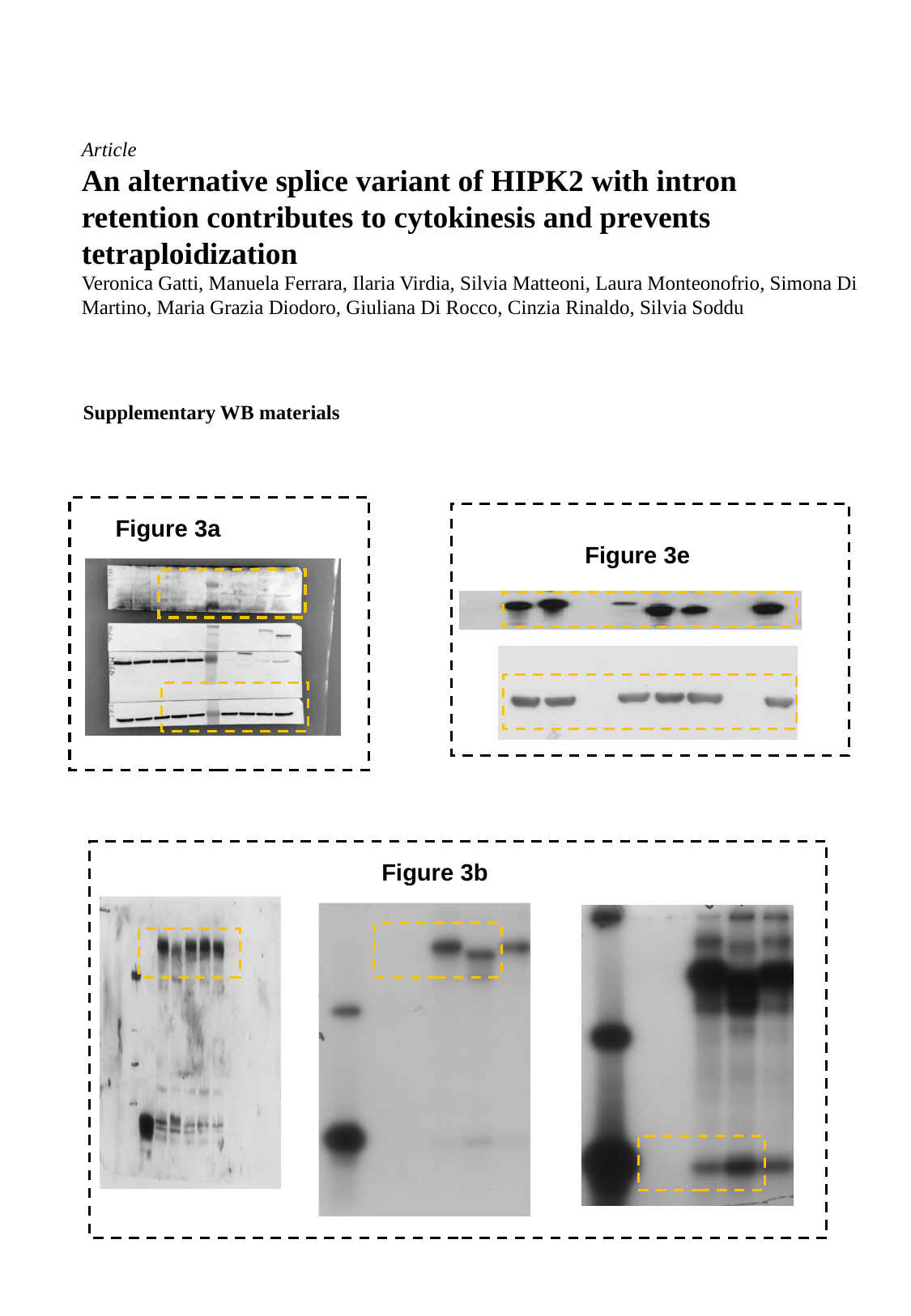

Article
An alternative splice variant of HIPK2 with intron retention contributes to cytokinesis and prevents tetraploidization
Veronica Gatti, Manuela Ferrara, Ilaria Virdia, Silvia Matteoni, Laura Monteonofrio, Simona Di Martino, Maria Grazia Diodoro, Giuliana Di Rocco, Cinzia Rinaldo, Silvia Soddu
Supplementary WB materials
Figure 3a
Figure 3e
Figure 3b
5m
GFP
FL
I13
D8

## Slide 2
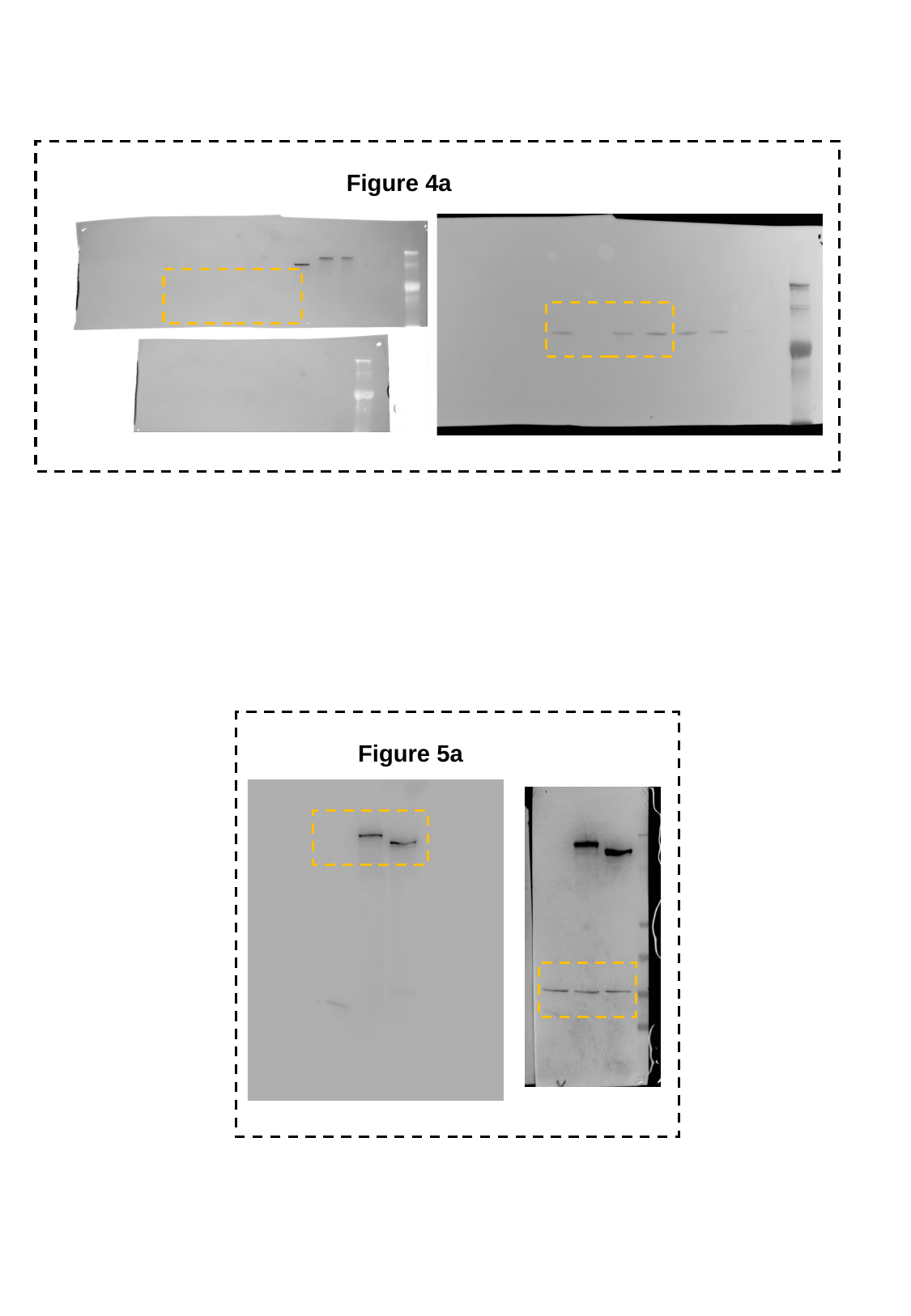

Figure 4a
Figure 5a

## Slide 3
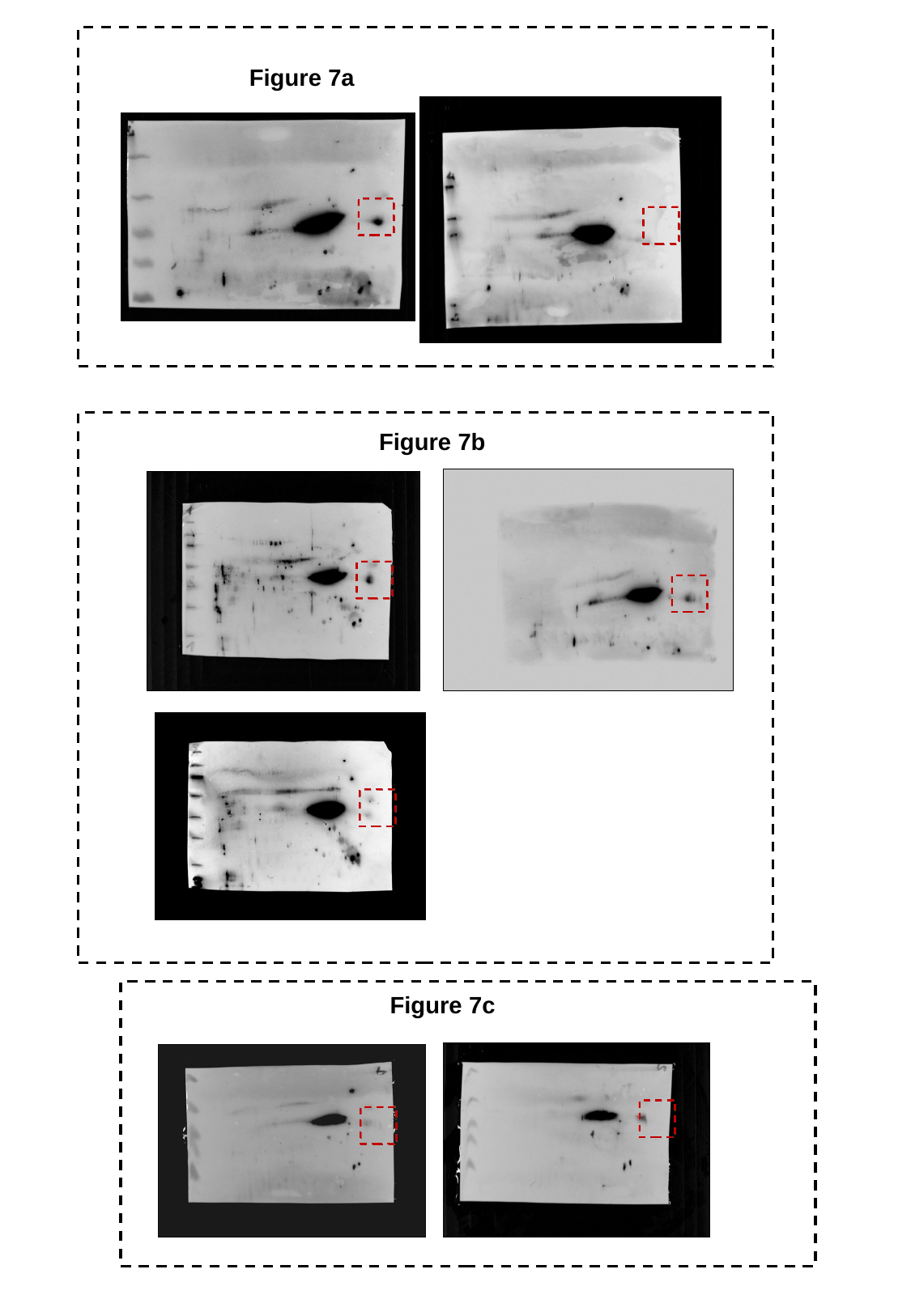

Figure 7a
Figure 7b
Figure 7c

## Slide 4
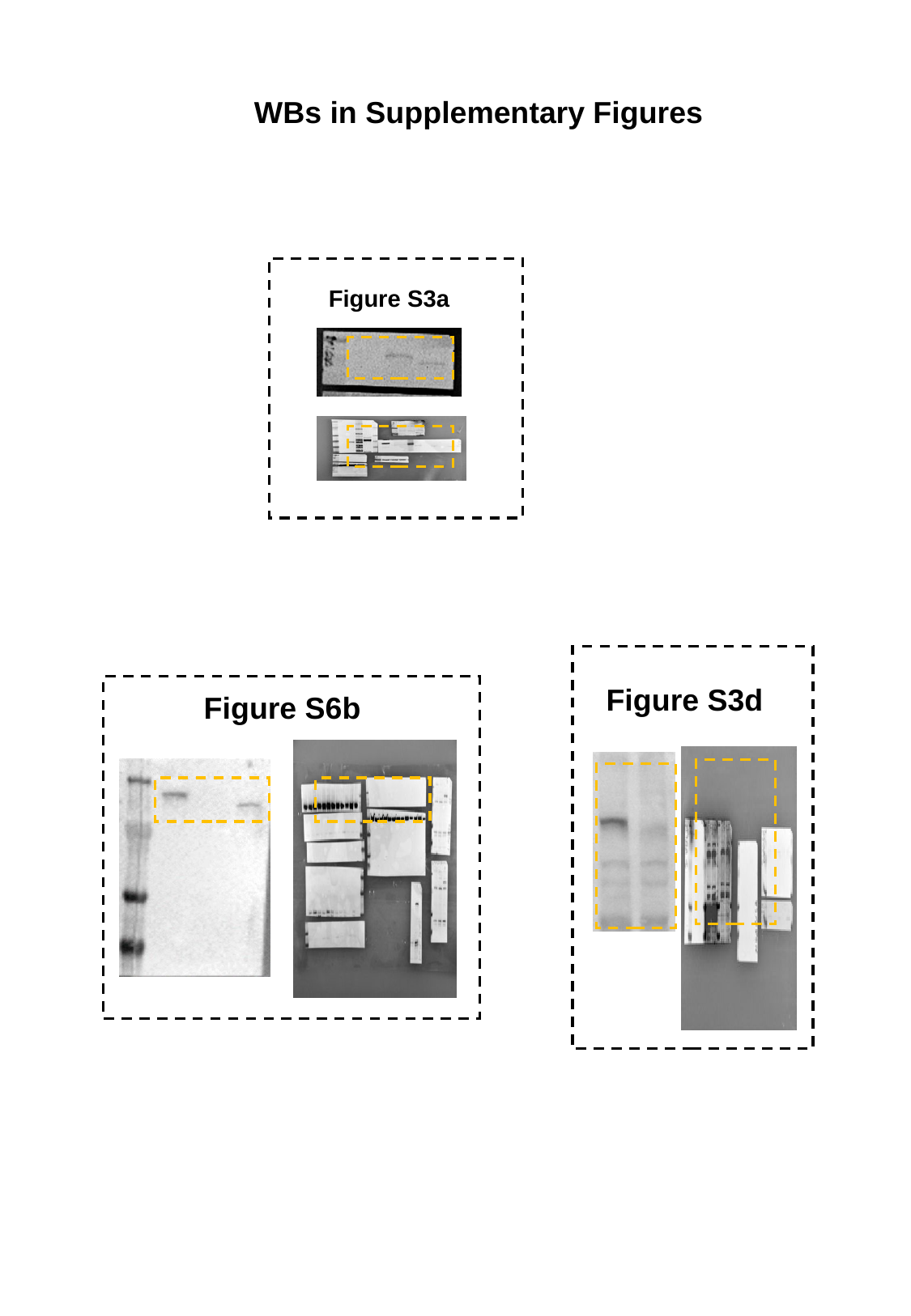

WBs in Supplementary Figures
Figure S3a
Figure S3d
Figure S6b
